# Antigenic imprinting in SARS‐CoV‐2

**DOI:** 10.1002/ctm2.923

**Published:** 2022-07-08

**Authors:** S. Momsen Reincke, Harald Prüss, Ian A. Wilson, Jakob Kreye

**Affiliations:** ^1^ German Center for Neurodegenerative Diseases (DZNE), Berlin Germany; ^2^ Department of Neurology and Experimental Neurology Charité—Universitätsmedizin Berlin Berlin Germany; ^3^ Department of Integrative Structural and Computational Biology The Scripps Research Institute La Jolla California USA; ^4^ The Skaggs Institute for Chemical Biology The Scripps Research Institute La Jolla California USA; ^5^ Department of Pediatric Neurology Charité—Universitätsmedizin Berlin Berlin Germany

**Keywords:** antigenic imprinting, SARS‐CoV‐2, vaccination strategies, variants of concern

## 1 IMMUNE EVASION OF SARS‐COV‐2 VARIANTS AND COUNTERMEASURES

Within weeks after the first detection of the Omicron strain, it became clear that this rapidly spreading and immune‐evasive variant of concern (VOC) severely reduces the protective efficacy of SARS‐CoV‐2 vaccines and resists most clinically approved monoclonal antibody (mAb) treatments. Multiple studies have shown that a third dose of the ancestral mRNA vaccines can restore neutralisation titres against Omicron to a level comparable to neutralisation of Delta after two doses.^1^ Nevertheless, the emergence of Omicron stimulated the development of mRNA vaccines based on the Omicron sequence that were designed to provide additional benefits when used as a booster or even as a primary vaccination strategy. However, in macaque studies that compared boosts with an ancestral wild‐type mRNA vaccine versus Omicron^2^ or Beta^3^ mRNA vaccines, no significant differences in neutralisation titres and disease protection were observed. Likewise, the first human studies with Beta‐ or Delta‐based mRNA boosts showed similar neutralisation titres among different variant booster regimes.^4^

## 2 WHY HAVE VOC‐BASED BOOSTERS SO FAR NOT SHOWN ADDITIONAL BENEFITS?

The lack of additional protection from VOC‐specific vaccines is likely related to the mode of action of booster immune responses. These responses are mainly shaped by the recall of memory B cells that have been elicited from previous exposures to similar antigens, a phenomenon called ‘antigenic imprinting’. Immunological imprints have been well studied in influenza infections, where birth‐year‐related first viral subtype exposure to influenza A virus (group 1 [H1N1, H2N2] or group 2 [H3N2]) imparts differential susceptibility to other potentially fatal influenza viruses, such as H5N1 (group 1) and H7N9 (group 2).^5^ While such imprints can be beneficial, neutral or detrimental, they have long‐term effects for antigen‐specific protection. Thus, its understanding may help to evaluate and improve vaccination strategies against a selected target. For SARS‐CoV‐2 immunity, evidence of immunological imprinting from other betacoronaviruses has been concluded by the observation that previous seasonal coronavirus infections have negative effects on the induction of immunoglobin G (IgG) and IgM against SARS‐CoV‐2.^6^ However, in the context of exposure to multiple SARS‐CoV‐2 variants, we are only beginning to understand the possible scope, effects and mechanisms of antigenic imprinting. A recent study provided the first insights from polyclonal antibody responses.^7^ The authors found that antibodies from non‐preimmunised individuals after Alpha or Delta variant infection preferentially bound the Alpha and Delta variant receptor‐binding domain (RBD), respectively. In contrast, antibodies from individuals who had been preimmunised with ancestral strain vaccinations and had breakthrough infections with the Alpha or Delta variant exhibited RBD variant binding patterns similar to those in individuals exposed to the ancestral strain only.^7^ While these data provide evidence for immunological imprinting from the initial variant exposure(s), an in‐depth mechanistic understanding and insights into preferential epitopes for B‐cell recall still remain unknown and require studies that investigate humoral responses to the VOCs at the resolution of monoclonal antibodies.

We recently published a study characterising monoclonal RBD antibody responses elicited by SARS‐CoV‐2 Beta variant infections in immunologically naïve subjects and compared these to antibodies elicited by the ancestral virus.^8^ We found that approximately half of the Beta‐elicited mAbs isolated from memory B cells did not bind the ancestral strain, while other mAbs potently neutralised Beta, the ancestral virus, and other VOCs. Cross‐protection between Beta and other VOCs, including Omicron, but not ancestral strains, could result from elicitation of mAbs to epitopes that are modified from or not as immunogenic in the ancestral virus. As this study was performed in SARS‐CoV‐2 naïve individuals, it does not mimic the immunologic situation of VOC boosters. However, it already indicates, that after immunisation with an antigenically distant SARS‐CoV‐2 variant, a notable portion of the elicited memory B cells can be variant‐specific and thus leave a specific imprint on which memory B cells are available for future recall with the same or variant antigen.

To better understand the functional role of antigenic imprinting in SARS‐CoV‐2 immunity against variants and possible implications for (booster) vaccination strategies, studies need to specifically investigate mAbs elicited in individuals who are subsequently exposed to different viral variant(s). The high incidences of Omicron breakthrough infections in vaccinated individuals provide an opportunity to study the recall of B cells in preimmunised individuals in a systematic manner. Recently, two studies were (pre)published dissecting the antibody response of such Omicron breakthrough infections. In the first, not yet peer‐reviewed preprint, Quandt et al.^9^ showed that Omicron breakthrough infections increase the frequency of pre‐existing memory B cells that recognise conserved epitopes rather than eliciting new B cells against Omicron‐specific epitopes, thereby highlighting the importance of antigenic imprinting in SARS‐CoV‐2. The serum neutralising activity was boosted not only against Omicron but also against previous SARS‐CoV‐2 VOCs as well as SARS‐CoV.

In the second study, Kaku et al.^10^ assessed the spike B‐cell response in ancestral mRNA‐vaccinated donors with Omicron breakthrough infection. During the acute phase, antibodies had a bias towards recognition and neutralisation of the ancestral SARS‐CoV‐2 strain, suggesting early activation of vaccine‐induced memory B cells. Interestingly, Omicron breakthrough infection led to a shift in B‐cell immunodominance, with B cells elicited after Omicron breakthrough infection more frequently targeting the RBD than the S2 subunit. The authors discuss that this finding may be a result of epitope masking of the previously dominant S2 subunit by pre‐existing serum antibodies that leads to a reduction in B‐cell access to the conserved S2 subunit compared to the more antigenically variable and subdominant RBD, a phenomenon that has similarly been described in malaria vaccinations.^11^ Another factor may be differential relative exposure or mobility of the RBD in variants, such as Omicron, that would affect how it is seen by the immune system.^12^

Both studies convincingly show that Omicron breakthrough infections predominantly activate pre‐existing cross‐reactive memory B cells, thereby confirming that antigenic imprinting plays a relevant role in SARS‐CoV‐2 immunity to viral variants. However, antigenic imprinting from ancestral strain mRNA vaccinations does not seem to impair the immune response against Omicron and thus so far does not raise any imprint‐related efficacy concerns about existing vaccination strategies. Nonetheless, there are still many open questions to be addressed. On the one hand, our current understanding of SARS‐CoV‐2 B‐cell recall is limited to single timepoint investigations on the monoclonal level and should be complemented with longitudinal investigations to mechanistically understand and track single antibody clones and their maturation specific to the variant's antigens. On the other hand, with the uncertainty regarding future variants, the role of antigenic imprinting in SARS‐CoV‐2 immunity may change quickly and drastically in the future, possibly with beneficial or detrimental net effects. Thus, in the evaluation of novel viral variants, the impact of immunological imprinting should be carefully monitored to understand its consequences and to fine‐tune immunisation strategies.
